# Hydroxytyrosol Ameliorates Endothelial Function under Inflammatory Conditions by Preventing Mitochondrial Dysfunction

**DOI:** 10.1155/2018/9086947

**Published:** 2018-04-18

**Authors:** Nadia Calabriso, Antonio Gnoni, Eleonora Stanca, Alessandro Cavallo, Fabrizio Damiano, Luisa Siculella, Maria Annunziata Carluccio

**Affiliations:** ^1^National Research Council-Institute of Clinical Physiology, Lecce, Italy; ^2^Department of Basic Medical Sciences, Neurosciences and Sense Organs, University of Bari “Aldo Moro”, Bari, Italy; ^3^Laboratory of Biochemistry and Molecular Biology, Department of Biological and Environmental Sciences and Technologies, University of Salento, Lecce, Italy

## Abstract

Mitochondria are fundamental organelles producing energy and reactive oxygen species (ROS); their impaired functions play a key role in endothelial dysfunction. Hydroxytyrosol (HT), a well-known olive oil antioxidant, exerts health benefits against vascular diseases by improving endothelial function. However, the HT role in mitochondrial oxidative stress in endothelial dysfunction is not clear yet. To investigate the HT effects on mitochondrial ROS production in the inflamed endothelium, we used an *in vitro* model of endothelial dysfunction represented by cultured endothelial cells, challenged with phorbol myristate acetate (PMA), an inflammatory, prooxidant, and proangiogenic agent. We found that the pretreatment of endothelial cells with HT (1–30 *μ*mol/L) suppressed inflammatory angiogenesis, a crucial aspect of endothelial dysfunction. The HT inhibitory effect is related to reduced mitochondrial superoxide production and lipid peroxidation and to increased superoxide dismutase activity. HT, in a concentration-dependent manner, improved endothelial mitochondrial function by reverting the PMA-induced reduction of mitochondrial membrane potential, ATP synthesis, and ATP5*β* expression. In PMA-challenged endothelial cells, HT also promoted mitochondrial biogenesis through increased mitochondrial DNA content and expression of peroxisome proliferator-activated receptor gamma coactivator 1-alpha, nuclear respiratory factor-1, and mitochondrial transcription factor A. These results highlight that HT blunts endothelial dysfunction and pathological angiogenesis by ameliorating mitochondrial function, thus suggesting HT as a potential mitochondria-targeting antioxidant in the inflamed endothelium.

## 1. Introduction

The vascular endothelium is a multifunctional organ critically involved in preserving vascular homeostasis through multiple functions including regulation of vascular tone and barrier, leukocyte trafficking, blood coagulation, nutrient and electrolyte uptake, and neovascularization of hypoxic tissue [1]. Chronic and degenerative diseases, such as cardiovascular diseases, are associated with alterations of endothelial physiological function, a condition termed endothelial dysfunction [2], characterized by reduced nitric oxide and increased reactive oxygen species (ROS) levels, endothelial inflammatory markers, and aberrant angiogenesis [3, 4]. One of the early manifestations of endothelial dysfunction is the dysregulation of mitochondrial function and biogenesis [5, 6]. Mitochondria, when deregulated, are either a major source or a target of oxidative stress, leading to a vicious circle. Unbalanced and extensive formation of mitochondrial ROS (mtROS) results in oxidative damage to many cellular components; this process in turn accelerates ROS production and generates mitochondrial dysfunction through alterations of mitochondrial membrane potential, ATP production, and mitochondrial biogenesis [7]. Since increased mtROS production appears to be a key event in altered endothelial functions and inflammatory angiogenesis involved in vascular pathologies [8], identification of mechanisms underlying mitochondrial dysfunction in endothelial cells may contribute to the development of improved approaches for vascular health. There is now a great interest to know whether natural dietary antioxidant compounds, reducing oxidative stress, can safeguard mitochondrial function in the vascular endothelium [9].

Hydroxytyrosol (HT), the major antioxidant phenolic compound present in olives and virgin olive oil, shows beneficial effects on chronic, inflammatory, and degenerative diseases [10, 11]. Most of the health effects of HT studied so far are connected with its ROS-scavenging property and with its ability to activate endogenous antioxidant systems [12–14], to blunt vascular inflammation and to improve endothelial function [15–18]. It has been previously shown that HT counteracted endothelial dysfunction by reducing endothelial inflammatory mediators including endothelial adhesion molecules and inflammatory cytokines [19, 20]. HT also reduced inflammatory angiogenesis, a key pathogenic process in cancer and in the development and vulnerability of atherosclerotic plaque, through inhibition of the proinflammatory enzyme cyclooxygenase-2, prostanoid production, and gelatinases, the matrix-degrading enzymes [21]. These effects were accompanied by a significant reduction in the activation of nuclear factor- (NF-) *κ*B, the redox-sensitive transcription factor, and in the production of intracellular ROS by endothelial NADPH oxidase [21, 22]. However, the potential role of HT in endothelial mtROS production under inflammatory conditions has not been examined yet.

In the present study, we analysed the HT effect on mtROS production and mitochondrial function in inflammatory angiogenesis. To this aim, we utilised a well-known *in vitro* model of human cultured vascular endothelial cells, challenged with phorbol myristate acetate (PMA), an inflammatory, prooxidant, and proangiogenic agent leading to endothelial dysfunction.

In this *in vitro* model, we monitored the HT effects on (i) pathological angiogenesis and mitochondrial oxidative stress; (ii) mitochondrial function by evaluating mitochondrial membrane potential and mitochondrial ATP production; and (iii) mitochondrial biogenesis through mitochondrial DNA content and the expression of factors coordinating the mitochondrial biogenesis, such as peroxisome proliferator-activated receptor gamma coactivator 1-alpha (PGC-1*α*), nuclear respiratory factor-1 (NRF-1), and mitochondrial transcription factor A (TFAM).

## 2. Materials and Methods

### 2.1. Materials

The materials for cell cultures were obtained from Gibco/BRL. Hydroxytyrosol (HT, 4-(2-hydroxyethyl)-1,2-benzenediol) was obtained from Cayman Chemical (Ann Arbor, MI), and PMA from Sigma-Aldrich (St. Louis, MO). Superoxide-sensitive probe MitoSOX Red, mitochondrial membrane potential probe JC-1, and CM-H2DCFDA probe were purchased from Molecular Probes. Primary antibodies against *β* subunit of human ATP synthase (ATP5*β*), PGC-1*α*, and NRF-1 and peroxidase-conjugated secondary antibody were purchased from Santa Cruz Biotechnology. Unless otherwise indicated, all other reagents were purchased from Sigma-Aldrich.

### 2.2. Cell Culture and Treatment

Human umbilical vein endothelial cells (HUVEC) were harvested, characterized, and maintained as previously described [21]. The human microvascular endothelial cell line (HMEC-1), obtained from Dr. Thomas J. Lawley, was cultured as described [21]. Confluent endothelial cells were shifted to medium supplemented with 3% foetal bovine serum (FBS) and subsequently treated with increasing concentrations of HT (0, 1, 10, and 30 *μ*mol/L) for 1 h and then stimulated with 10 nmol/L PMA. Stock solution of HT (1 mg/mL) was made in absolute ethanol, and stock solution of PMA (300 *μ*mol/L) in DMSO. As a vehicle control, HUVEC were incubated with an appropriate amount of each solvent (<0.025% *v*/*v*). The used solvents had no effects on any of the parameters measured. Cellular toxicity was checked by a variety of techniques including cell count, protein content, trypan blue exclusion, and MTT (3-(4,5-dimethylthiazolyl-2)-2,5-diphenyltetrazolium bromide) assays. In preliminary experiments aimed at evaluating phytochemical toxicity, treatment of HUVEC or HMEC-1 with concentrations of HT up to 30 *μ*mol/L for 24 h did not produce any sign of toxicity.

### 2.3. Detection of Cellular ROS and Mitochondrial Superoxide Production

Cellular ROS formation was assessed using a carboxy-2′,7′-dichlorofluorescein diacetate (CM-H2DCFDA) probe. CM-H2DCFDA freely permeates the plasma membrane and is hydrolyzed in the cytosol to form the DCFH carboxylate anion [23, 24]. Oxidation results in the formation of fluorescent DCF, which is maximally excited at 495 nm and emitted at 520 nm [24]. HUVEC at confluence were incubated with HT (0, 1, 10, and 30 *μ*mol/L) for 1 h, then loaded with the probe CM-H2DCFDA (10 *μ*mol/L) for 45 min at 37°C, in the dark. Following the incubation, monolayers were gently washed in PBS twice, then stimulated with 10 nmol/L PMA in phenol red-free medium for 0–90 min and monitored by spectrofluorimetric analysis.

Mitochondrial superoxide production was assessed using MitoSOX Red, a mitochondria-targeting fluorescent probe, according to the manufacturer's instructions. MitoSOX Red exhibits fluorescence after oxidation by superoxide anion (excitation 510 nm, emission 580 nm) [25]. HUVEC monolayers were treated with HT (0, 1, 10, and 30 *μ*mol/L) for 1 h, then loaded with the probe MitoSOX Red (5 *μ*mol/L) for 20 min at 37°C in the dark and next stimulated with 10 nmol/L PMA in phenol red-free medium for 0–90 min. MitoSOX Red fluorescence was measured by spectrofluorimetric analysis. Alternatively, for long-term stimulation (10 nmol/L PMA for 16 h), HUVEC were incubated at 37°C in 3% FBS-containing phenol red-free medium in the dark before measurement of MitoSOX Red fluorescence by spectrofluorimetry or by fluorescence microscopy.

### 2.4. Cell Migration and Tube Formation Assay

HUVEC were cultured in 6-well plates until confluence and then incubated with increasing concentrations of HT (0, 1, 10, and 30 *μ*mol/L) or electron transport chain inhibitors, rotenone or antimycin A (1 *μ*mol/L), for 1 h. Afterwards, a scratch wound was performed with a sterile microtip under standard conditions. After washing with PBS to remove detached cells, a first series of photos were taken by an attached digital output Canon Powershot S50 camera (0 h). Monolayers were then stimulated with 10 nmol/L PMA for 16 h in serum-free medium containing 0.1% human serum albumin, a condition that allows cell survival but not cell proliferation. Monolayers were then washed and again photographed (16 h). Cell repair of the wound was determined by measuring the width (*μ*m) of the denuded area along the scratch (at five different levels) using the Optimas Image analysis software (Media Cybernetics, Pleasanton, CA). The formation of vascular-like structures by endothelial cells was assessed on the growth factor-reduced basement membrane matrix “Matrigel” (11.1 mg/mL; Becton Dickinson Biosciences, Bedford, MA) as previously described [21]. The bottoms of 24-well culture plates were coated with Matrigel (50 *μ*L per well) diluted at 1 : 2 with M199 medium. After gelatination at 37°C for 30 min, gels were overlaid with 500 *μ*L of 2% FBS-containing M199 medium containing 4 × 10^4^ cells per well. The media were supplemented with 10 nmol/L PMA in the absence or presence of 1–30 *μ*mol/L HT or rotenone or antimycin A (1 *μ*mol/L) for 1 h and then incubated for further 16 h at 37°C. Tube formation was monitored by inverted phase-contrast microscopy (Leica, Wetzlar, Germany), and pictures (×100 magnification) were taken by an attached digital output Canon Powershot S50 camera. Tubule branching points were counted in three randomly selected fields per well and were averaged.

### 2.5. Assessment of Mitochondrial Membrane Potential

Mitochondrial membrane potential (MMP) was assessed as described previously [26] using 5,5′,6,6′-tetrachloro-1,1′,3,3′-tetraethylbenzimidazol-carbocyanine iodine (JC-1), a cationic dye that exhibits MMP-dependent accumulation and formation of red fluorescent J-aggregates in mitochondria. Each set of samples included a positive control for mitochondrial depolarization (HUVEC or HMEC-1 treated with 1 *μ*mol/L carbonyl cyanide p-(tri-fluromethoxy)phenyl-hydrazone (FCCP)) and hyperpolarization (HUVEC or HMEC-1 treated with 1 *μ*g/mL oligomycin). Fluorescence was determined by a fluorimeter (Fluoroskan II, Labsystem, Helsinki, Finland) using excitation at 488 nm. The JC-1 monomer (green) and the J-aggregates (red) were detected at 530 nm and 590 nm emission, respectively. MMP is evaluated as the red-to-green fluorescence intensity ratio.

### 2.6. Lipid Peroxidation and Superoxide Dismutase Measurements

The level of cellular lipid peroxidation was determined through the formation of thiobarbituric acid-reactive species (TBARS) as reported in [27] following the method of Esterbauer and Cheeseman [28]. Briefly, malondialdehyde (MDA), a by-product of lipid peroxidation, forms an adduct with thiobarbituric acid (TBA) which was measured colorimetrically using an MDA equivalent standard. Butylated hydroxytoluene was added to each test sample to prevent further lipid oxidation during sample processing and the TBA reaction. The MDA production, expressed as nmol produced/mg protein, was followed spectrophotometrically at 533 nm. The superoxide dismutase (SOD) activities were determined with the Fluka analytical assay kit using a spectrophotometer Victor™ X (PerkinElmer) at *λ*  =  440 nm. The Cu/ZnSOD and the mitochondrial MnSOD activities were assayed, without or with KCN (4 mmol/L), using the ability to inhibit the reduction of WST-1 [2-(4-Iodophenyl)-3-(4-nitrophenyl)-5-(2,4-disulfophenyl)-2H-tetrazolium monosodium salt] by superoxide anions generated by the xanthine/xanthine oxidase method. One unit of SOD activity was defined as the amount of the enzyme causing half maximum inhibition of WST-1 reduction.

### 2.7. Evaluation of the Mitochondrial ATP Synthase Activity

Mitochondrial ATP synthesis was measured spectrophotometrically as described [29]. Briefly, 1 × 10^6^ cells were resuspended in 1 mL of buffer containing 10 mmol/L HEPES (pH 7.4), 150 mmol/L NaCl, 1 mmol/L K-EDTA, 20 mmol/L glucose, 2 mmol/L MgCl_2_, 5 U/mL hexokinase, 300 *μ*mol/L Ap5A, and 25 mmol/L KH_2_PO_4_. Endothelial cells were incubated, under stirring for rotation, in the absence or in the presence of oligomycin for 30 minutes. Then, the reaction was stopped with 3% HClO_4_, and the cells were centrifuged in Eppendorf tubes at 100 ×g for 4 minutes. To the supernatant (500 *μ*L), 500 *μ*L of buffer containing 1 mmol/L MgCl_2_, 150 mmol/L Tris/HCl (pH 7.4), and 7 U/mL glucose-6-phosphate dehydrogenase was added. The reaction was started by adding 1 mmol/L NADP, and the reduction of the coenzyme was followed spectrophotometrically (Beckman Coulter DU 800), at 360/374 nm with an *ɛ* = 2.1 mmol/L^−1^·cm^−1^.

### 2.8. Western Blot Analysis

Total cell extract was prepared as previously described [22]. After protein content determination, the cell lysate was separated using NuPAGE Bis-Tris precast 10% polyacrylamide gels under reducing conditions (Invitrogen, Carlsbad, CA, USA). Resolved proteins were transferred onto nitrocellulose sheets (Amersham, Freiburg, Germany), and the resulting membranes were saturated with a 5% blocking agent (Amersham) in Tris-buffered saline (TBS, 20 mmol/L Tris (pH 7.6) and 132 mmol/L NaCl) for 1 h at room temperature. Membranes were then incubated overnight at 4°C with primary antibodies against human ATP5*β*, PGC-1*α*, NRF-1, TFAM, and *β*-actin, followed by a horseradish peroxidase-conjugated secondary antibody. The enhanced chemiluminescence (ECL) method (Amersham) was used to reveal positive bands, according to the manufacturer's instructions. Bands were analysed quantitatively using the Scion Image Alpha 4.0.3.2 software (Scion Corporation).

### 2.9. Quantitative Reverse Transcription-Polymerase Chain Reaction Analysis

HUVEC were treated with HT (0, 1, 10, and 30 *μ*mol/L) for 1 h and then stimulated with 10 nmol/L PMA for 16 h. Total RNA was isolated by using the TRIzol reagent (Invitrogen) according to the manufacturer's protocol. For quantitative polymerase chain reaction, total RNA (2 *μ*g) was converted into first-strand cDNA by using the High-Capacity cDNA Reverse Transcription Kit (Applied Biosystems, Monza, Italy). The quantitative RT-PCR was performed in the Bio-Rad Biosystems CFX384 Touch Real-Time PCR Detection System, by using SYBR Green PCR Master Mix. The human cDNA fragments were amplified using primers synthesized by Sigma Genosys and reported in Table 1. We explored the expression of the following genes: *ATP5β*, *PGC-1α*, *NRF-1*, *TFAM*, *TNF-α*, *IL-1β*, *VCAM-1*, and *ICAM-1*. The quantifications were performed using the efficiency-adjusted ΔΔCT method (CFX Manager), with Gapdh/36B4 as an internal control.

### 2.10. Determination of the Mitochondrial DNA Copy Number

Total DNA from endothelial cells was obtained by phenol/chloroform extraction. Real-time quantitative PCR (qPCR) was performed to quantify mitochondrial DNA (mtDNA) content. MtDNA level was expressed as the ratio of mtDNA (D-loop) to nuclear DNA (Gapdh) quantity. The primers used for D-loop and Gapdh are reported in Table 1.

### 2.11. Statistical Analysis

Values were expressed as mean ± SD for the number of experiments indicated in the legends to the figures. Differences between two groups were determined by unpaired Student's *t*-test. Multiple comparisons were performed by one-way analysis of variance (ANOVA), and individual differences were then tested by Fisher's protected least significant difference test, after the demonstration of significant intergroup differences by ANOVA. Differences between means from at least three independent experiments with *p* < 0.05 were considered statistically significant.

## 3. Results

### 3.1. Hydroxytyrosol Inhibits PMA-Induced Angiogenic Response by Reducing Mitochondrial Superoxide Production

In previous studies, we characterized a model of endothelial dysfunction constituted by HUVEC challenged with PMA, an inflammatory and proangiogenic agonist able to induce endothelial activation and inflammatory angiogenesis [20, 21, 30]. In the same works, we have shown that HT reduced endothelial dysfunction [20, 21] by decreasing intracellular oxidative stress and NF-*κ*B activation, a pivotal regulator of inflammatory gene expression.

**Figure 1:**
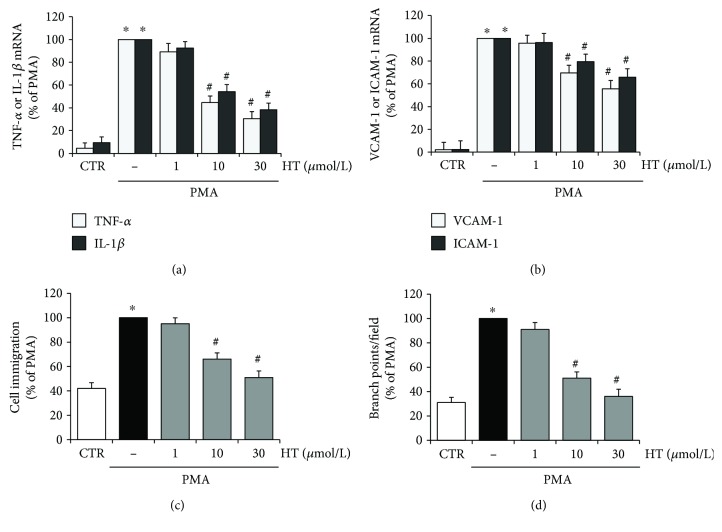
HT effects on PMA-induced endothelial dysfunction. HUVEC were pretreated with HT (1–30 *μ*mol/L) or vehicle (control, CTR) for 1 h and stimulated by PMA (10 nmol/L) for 16 h; then, TNF-*α* and IL-1*β* (a) or VCAM-1 and ICAM-1 (b) mRNA levels were determined by quantitative RT-PCR. HUVEC were pretreated with HT (1–30 *μ*mol/L) for 1 h; afterwards, a scratch wound was performed and monolayers were stimulated by 10 nmol/L PMA for 16 h. Cell migration was monitored under phase-contrast microscopy and quantified (c). HUVEC were plated onto a 3-dimensional collagen gel (Matrigel) surface, pretreated with HT (1–30 *μ*mol/L), for 1 h, and then stimulated with 10 nmol/L PMA for 16 h. Tube formation was monitored under phase-contrast microscopy and reported as branch points per field (d). ^∗^ *p* < 0.05 versus CTR; ^#^ *p* < 0.05 versus PMA alone.

In the present study, we evaluated the protective effect of HT on endothelial dysfunction by analysing inflammatory and angiogenic response in PMA-triggered endothelial cells (Figure 1). Figure 1 shows that HT (1–30 *μ*mol/L), in a concentration-dependent manner, reduced the expression of the PMA-stimulated inflammatory cytokines, tumor necrosis factor- (TNF-) *α* and interleukin- (IL-) 1*β* (Figure 1(a)), as well as the endothelial adhesion molecules, vascular cell adhesion molecule- (VCAM-) 1 and intercellular adhesion molecule- (ICAM-) 1 (Figure 1(b)). Figure 1 also shows that HT pretreatment reduced the PMA-induced angiogenic response (Figures 1(c) and 1(d)). The new vessel formation occurs through a series of steps, including endothelial cell migration and morphological differentiation/reorganization of endothelial cells into a three-dimensional tubular structure [31]. Therefore, we analysed the endothelial cell migration by scratch wound healing and capillary-like tube formation by the Matrigel assay. As shown in the bar graph (Figure 1(c)), the HT pretreatment inhibited, in a concentration-dependent manner, the migration of endothelial cells stimulated by PMA. Moreover, HT suppressed the PMA-challenged endothelial angiogenic activity by decreasing the capillary-like tube formation on Matrigel, as documented by the reduced number of branch points, shown in bar graph quantification (Figure 1(d)). The HT inhibitory effects were significantly evident at 10 *μ*mol/L and reached maximum inhibition at 30 *μ*mol/L.

Since increased mtROS production appears to be a critical event in endothelial dysfunction [8], in the present study, we deepened the HT effects on superoxide production and mitochondrial function in inflammatory angiogenesis.

**Figure 2:**
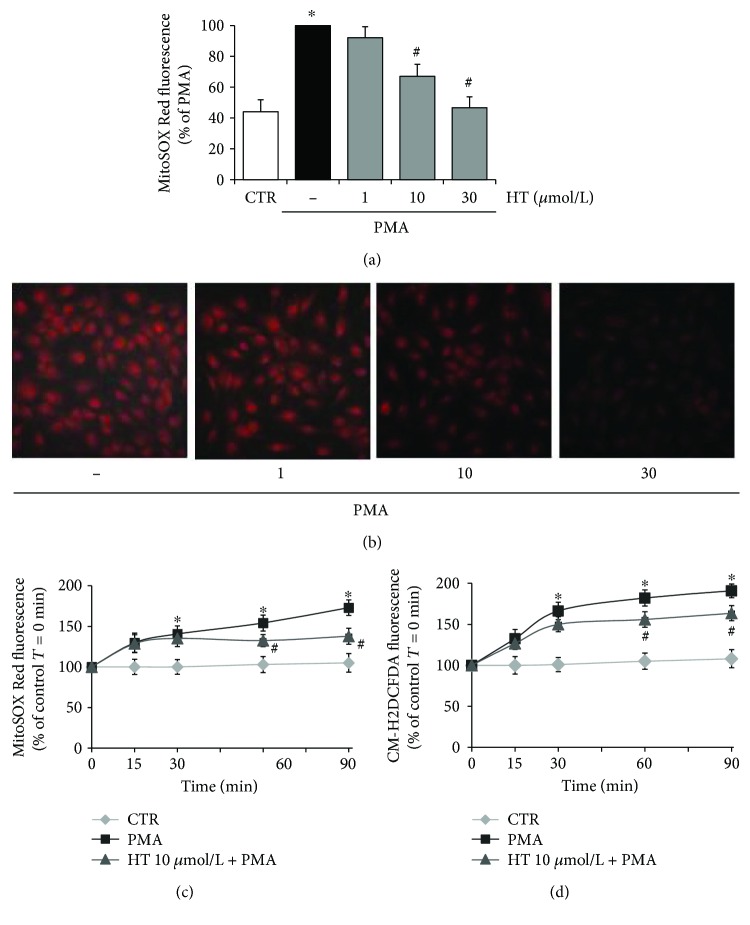
Effects of HT on PMA-induced intracellular ROS and mitochondrial superoxide production. HUVEC were pretreated with HT (1–30 *μ*mol/L) or vehicle (control, CTR) for 1 h and loaded by CM-H2DCFDA or MitoSOX Red as described in Materials and Methods, then stimulated with 10 nmol/L PMA for 16 h (a, b) and for the indicated times (c, d), and next fluorescence levels were evaluated by a fluorescence plate reader (a), fluorescence microscope (b), or fluorimeter (c, d). Data represent three independent experiments performed in triplicate and are expressed as means ± SD. ^∗^ *p* < 0.05 versus CTR; ^#^ *p* < 0.05 versus PMA alone.

In Figure 2(a), we reported the HT effects on mtROS in PMA-triggered endothelial cells showing that PMA stimulation greatly increased superoxide generation in HUVEC, as evaluated by increased MitoSOX Red fluorescence, resulting from the oxidation of MitoSOX Red by mitochondrial superoxide. HT pretreatment reduced the PMA-induced superoxide production: 10 *μ*mol/L HT decreased superoxide by about 33% and 30 *μ*mol/L HT lowered it to the control levels (Figure 2(a)). The inhibitory effect of HT on PMA-induced mitochondrial superoxide production was confirmed by fluorescence microscopy imaging (Figure 2(b)). HUVEC challenged with PMA showed bright red fluorescence, which was markedly blunted after HT treatment in a concentration-dependent manner (Figure 2(b)). In addition, we investigated the temporal effects of HT on mtROS production in HUVEC challenged with PMA for short times (0–90 min).  Figure 2(c) shows that PMA boosted mtROS already at 30 min. HT at 10 *μ*mol/L, the lowest effective concentration against endothelial inflammation and dysfunction, significantly blunted, at 60 min, MitoSOX Red fluorescence, suggesting an early role of mtROS in PMA-triggered endothelial cells. The kinetics of mtROS production followed a trend similar to that of cytosolic ROS, determined by CM-H2DCFDA fluorescence (Figure 2(d)). In our model system, the levels of nitric oxide, a marker of endothelial dysfunction, were also evaluated. Results showed no difference in nitric oxide levels either after PMA stimulation or after HT treatment (Supplementary Figure 1). These results highlight a crucial role of mtROS both in PMA-induced endothelial dysfunction and in HT protective action.

Noteworthy, the inhibitory actions of HT occurred only under proinflammatory conditions induced by PMA treatment and in the absence of any toxicity as determined by the MTT assay and protein content and cellular counts (data not shown). Indeed, HT treatment did not display significant effects in HUVEC under basal conditions on mitochondrial ROS production, cell migration, and capillary-like formation (data not shown).

**Figure 3:**
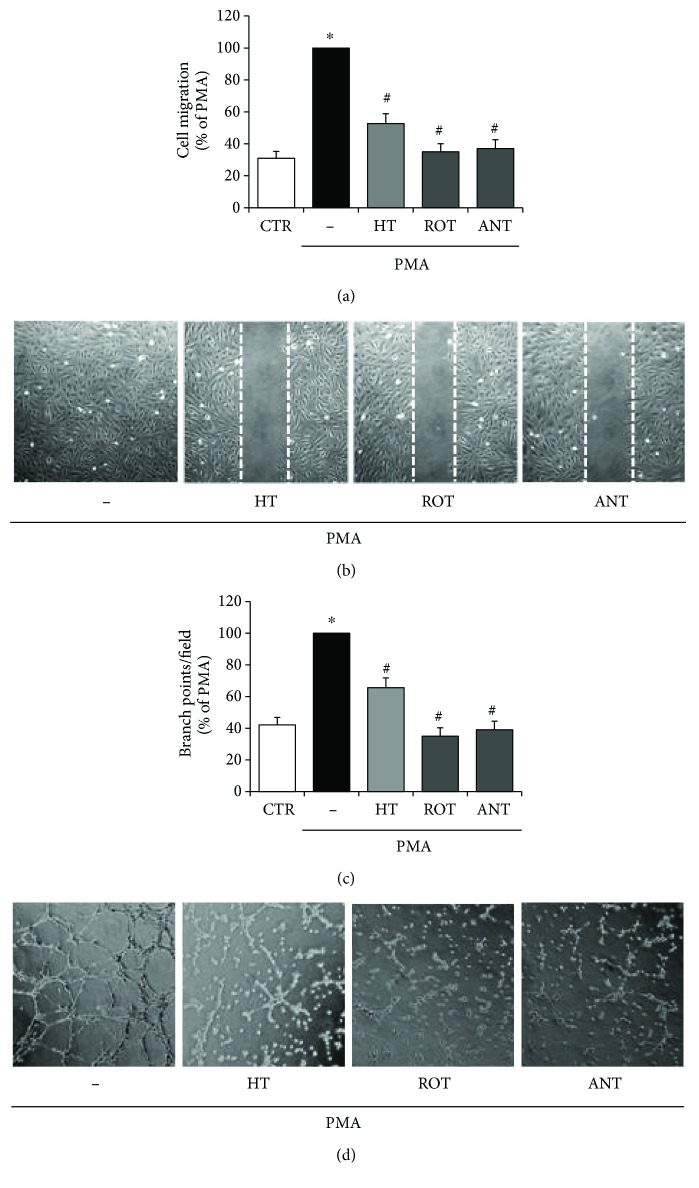
HT effects on PMA-induced mitochondrial superoxide production in cell migration and in inflammatory angiogenesis. HUVEC were pretreated with HT (10 *μ*mol/L) and rotenone or antimycin A (1 *μ*mol/L) for 1 h; afterwards, a scratch wound was performed and monolayers were stimulated by 10 nmol/L PMA for 16 h. Cell migration was quantified and monitored under phase-contrast microscopy (a, b). HUVEC were plated onto a 3-dimensional collagen gel (Matrigel) surface, pretreated with HT (10 *μ*mol/L) and rotenone or antimycin A (1 *μ*mol/L) for 1 h, and then stimulated with 10 nmol/L PMA for 16 h. Tube formation was monitored under phase-contrast microscopy, photographed, and analysed (c, d). Images are representative of cell migration (b), and capillary-like tube formation is reported as branch points per field (d) in PMA-stimulated endothelial cells (×100 magnification). Data are representative of three independent experiments, expressed as means ± SD, and presented as percentage of PMA-stimulated endothelial cells. Each experiment consisted of four replicates for each condition. ^∗^ *p* < 0.05 versus CTR; ^#^ *p* < 0.05 versus PMA alone.

Finally, to analyse the role of superoxide produced by mitochondria in inflammatory angiogenic response, HUVEC were pretreated with rotenone or antimycin A, inhibitors of the mitochondrial complexes I and III, main sites of ROS production in the electron transport chain, respectively. The effects of the two inhibitors on PMA-induced endothelial cell migration and tubule formation were evaluated. As shown in the bar graph (Figure 3(a)) and in representative scratch wound healing images (Figure 3(b)), the pretreatment with rotenone or antimycin A inhibited the migration of endothelial cells stimulated by PMA. Moreover, both the inhibitors suppressed the PMA-challenged endothelial angiogenic activity by decreasing the capillary-like tube formation on Matrigel, as documented by the reduced number of branch points, shown in representative images (Figure 3(d)) and in bar graph quantification (Figure 3(c)). These inhibitory effects of rotenone or antimycin A point out the key role of mtROS in aberrant angiogenesis under inflammatory conditions. In HUVEC under basal unstimulated conditions, rotenone and antimycin A alone did not cause substantial effects on mtROS production, cell migration, and capillary-like formation.

In addition to macrovascular endothelial cells (HUVEC), microvascular endothelial cells (HMEC-1) were used in comparative experiments and similar results were obtained (data not shown).

**Figure 4:**
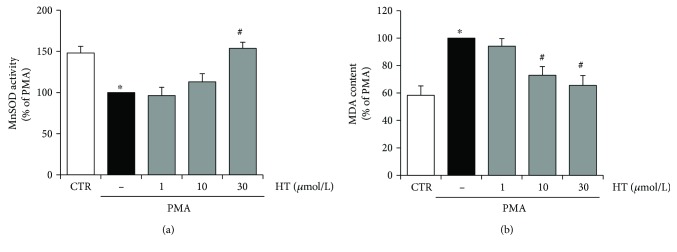
HT effects on SOD activity and oxidative damage in stimulated endothelial cells. HUVEC were pretreated with HT (1–30 *μ*mol/L) for 1 h and then stimulated with PMA for further 16 h; afterwards, the activity of MnSOD (a) and lipid peroxidation, followed by MDA production (b), were evaluated. Data are representative of three independent experiments (mean ± SD) and expressed as percentage of PMA-stimulated endothelial cells. ^∗^ *p* < 0.05 versus CTR; ^#^ *p* < 0.05 versus PMA alone.

To further analyse the protective role of HT in endothelial oxidative stress under inflammatory conditions, the HT effects on SOD activities and oxidative damage were examined. We found that PMA reduced MnSOD activity, which was rescued by 30 *μ*mol/L HT pretreatment (Figure 4(a)). The uncontrolled production of cellular ROS induced, among others, direct damages of cellular lipids, the so-called lipid peroxidation, which was assayed by measuring the level of its end product MDA [32]. PMA stimulation increased cellular MDA levels by about 40%, with respect to control unstimulated cells, thus confirming an enhancement of cellular oxidative stress (Figure 4(b)). Pretreatment with HT reduced PMA-induced lipid peroxide production by about 27% at 10 *μ*mol/L and 35% at 30 *μ*mol/L (Figure 4(b)).

The present findings reveal an important role of mtROS in inflammatory angiogenesis and show that HT treatment can improve endothelial function by decreasing mitochondrial superoxide production and oxidative damage.

### 3.2. Hydroxytyrosol Prevents Mitochondrial Oxidative Dysfunction in the Inflamed Endothelium

**Figure 5:**
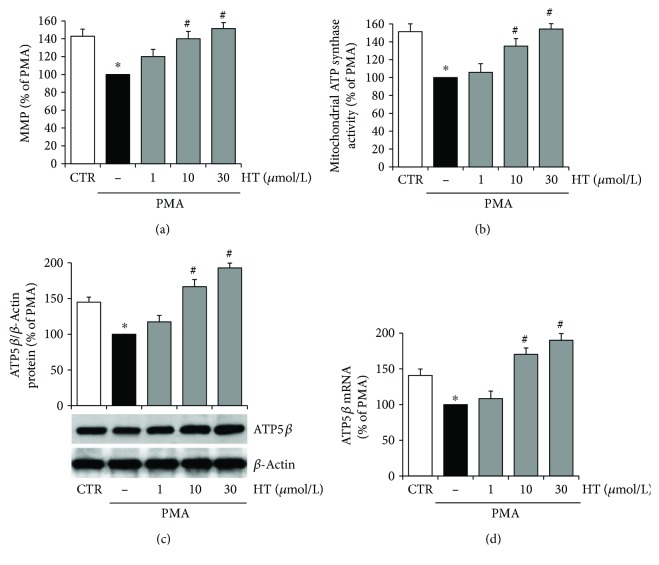
HT improves mitochondrial function in PMA-stimulated endothelial cells. HUVEC were pretreated with HT (1–30 *μ*mol/L) for 1 h and then stimulated with PMA (10 nmol/L) for further 16 h. After treatments, MMP was assayed by using JC-1 staining and evaluated by using a fluorescence plate reader (a). Mitochondrial oligomycin-sensitive ATP synthesis was measured in endothelial cells incubated in the absence (−) or in the presence of HT (1–30 *μ*mol/L) (b). The expression of *β* subunit of ATP synthase was evaluated at protein (c) and mRNA levels (d) by Western blotting or quantitative RT-PCR, respectively. ATP5*β* protein expression was normalized to *β*-actin, and ATP5*β* mRNA amount was normalized to Gapdh mRNA. Data are representative of four independent experiments (mean ± SD), each consisting of four replicates for each condition, and expressed as percentage of PMA-stimulated endothelial cells. ^∗^ *p* < 0.05 versus CTR; ^#^ *p* < 0.05 versus PMA alone.

There is increasing evidence that mitochondrial alterations are implicated in vascular endothelial inflammation and angiogenesis [8]. Since mitochondrial membrane potential (MMP) is an important indicator of mitochondrial function in situ, we monitored the MMP in PMA-challenged endothelial cells by using the JC-1 assay.  Figure 5(a) shows that PMA stimulation induced a significant MMP decrease, with respect to unstimulated control cells. Pretreatment with HT reverted the PMA-induced depolarization of the mitochondrial membrane with an effect already significant at 10 *μ*mol/L (Figure 5(a)).

MMP drives the synthesis of ATP; therefore, changes in the membrane potential can affect mitochondrial ATP synthesis. We assessed whether in endothelial cells the MMP, reduced by PMA, was connected with changes in mitochondrial ATP synthesis, and we analysed the effects of HT pretreatment. In accordance with MMP alterations (Figure 5(a)), we observed that PMA challenge reduced by about 34% the fraction of ATP produced by endothelial mitochondrial FoF1-ATP synthase (Figure 5(b)). In detail, in unstimulated endothelial cells, the amount of ATP synthesized by the FoF1-ATP synthase was 10.3 ± 1.3 nmol of ATP formed/min/1 × 10^6^ cells; meanwhile, in PMA-challenged cells, it was lowered to 6.8 ± 1.1 nmol of ATP formed/min/1 × 10^6^ cells. HT pretreatment reverted the PMA-reduced mitochondrial ATP production in a concentration-dependent manner, increasing it by about 35% versus PMA at 10 *μ*mol/L HT and reaching the control values at 30 *μ*mol/L HT. Since it is well known that the activity of ATP synthase is influenced by the expression of the enzyme catalytic subunit ATP5*β*, we investigated the ATP5*β* protein and mRNA levels. We found that PMA significantly reduced the expression of ATP5*β* protein in endothelial cells, while HT pretreatment reverted PMA-reduced ATP5*β* expression already at 10 *μ*mol/L HT and caused a further increase in the protein content at 30 *μ*mol/L HT (Figure 5(c)). Consistently with ATP5*β* protein levels, the ATP5*β* mRNA abundance significantly decreased following PMA stimulation with respect to unstimulated control cells, while preincubation with 10 *μ*mol/L HT reverted PMA-reduced ATP5*β* mRNA amount to the unstimulated control level (Figure 5(d)).

These results highlight that HT treatment was able to improve endothelial mitochondrial function under inflammatory conditions, by counteracting the decrease in MMP as well as in ATP synthesis and ATP5*β* expression.

### 3.3. Hydroxytyrosol Promotes Mitochondrial Biogenesis in the Inflamed Endothelium

**Figure 6:**
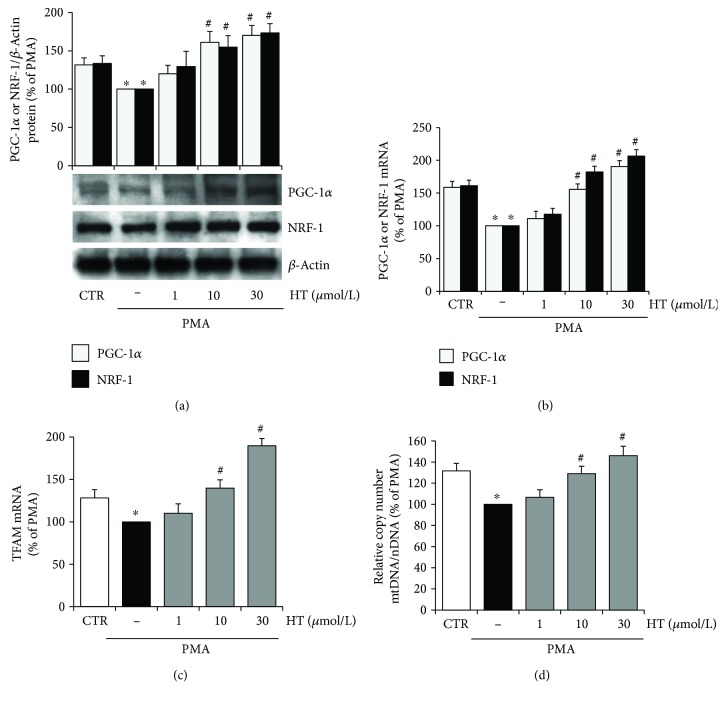
HT increases mitochondrial biogenesis in stimulated endothelial cells. HUVEC were pretreated with HT (1–30 *μ*mol/L) for 1 h and then stimulated with PMA for further 16 h. The expression of mitochondrial biogenesis factors was evaluated by Western blotting (a), and the corresponding mRNA levels were assessed by quantitative RT-PCR (b, c). DNA was isolated, and quantitative real-time PCR was used to determine nuclear DNA (nDNA) and mitochondrial DNA (mtDNA) contents. The mtDNA content was expressed as the ratio of the mtDNA copy number to the nDNA copy number (mtDNA/nDNA) (d). Data are representative of four independent experiments (mean ± SD) and expressed as percentage of PMA-stimulated endothelial cells. ^∗^ *p* < 0.05 versus CTR; ^#^ *p* < 0.05 versus PMA alone.

To determine whether in PMA-challenged endothelial cells the observed improvement in mitochondrial function promoted by HT was associated with increased mitochondrial biogenesis, the mtDNA content and the expression of PGC-1*α*, NRF-1, and TFAM, which play a pivotal role in this process, were investigated. Western blotting analysis and real-time qRT-PCR revealed that the expression of the mitochondrial biogenesis factors, PGC-1*α* and NRF-1, was significantly reduced by PMA treatment (Figures 6(a) and 6(b)). HT preincubation reverted the PMA-reduced expression of NRF-1 and PGC-1 with a significant effect at 10 *μ*mol/L, at both protein (Figure 6(a)) and mRNA levels (Figure 6(b)). PMA significantly reduced also the abundance of TFAM mRNA, which was increased by HT treatment (Figure 6(c)). Finally, the mtDNA copy number, which is a critical determinant of overall mitochondrial health, was analysed. To this aim, mtDNA and nuclear DNA (nDNA) were measured by real-time qPCR. The mtDNA copy number was expressed as the ratio of mtDNA (D-loop) to nDNA (Gapdh). PMA stimulation of endothelial cells decreased the mtDNA copy number with respect to unstimulated control cells. HT treatment reverted the PMA reduction of the mtDNA copy number already at 10 *μ*mol/L with a further increase at 30 *μ*mol/L (Figure 6(d)).

The present findings unveil that HT can improve endothelial function by promoting mitochondrial biogenesis under inflammatory conditions.

## 4. Discussion

HT, the major phenolic compound present in olives and virgin olive oil, exerts anti-inflammatory and antiangiogenic function through inhibition of intracellular ROS levels and NF-*κ*B activation in the inflamed endothelium [20, 21]. However, the role of HT in mtROS production in inflammatory angiogenic response has not been to date clarified.

In the present study, we show that the endothelial protective effect of HT occurs by inhibiting the expression of inflammatory cytokines and endothelial adhesion molecules and that HT prevents inflammatory angiogenesis by reducing mitochondrial superoxide production and by improving mitochondrial function and biogenesis.

At low concentrations, ROS could behave as proangiogenic signalling molecules in endothelial cells, but at elevated levels, ROS could cause endothelial cell dysfunction and pathological angiogenesis [33–35]. In vascular endothelial cells, mitochondria, far from being simply ATP-producing organelles, also play a key role in cell signalling through mtROS production [36, 37]. High levels of mtROS alter normal ROS signalling and mitochondrial functions leading to endothelial dysfunction and ultimately to the development of cardiovascular disease [38]. In vascular endothelial cells, the mitochondrial electron transport chain represents one of the major sites of ROS production. Although most electrons through the chain redox gradient reach at the end complex IV, they prematurely can react with oxygen, at the level of complexes I and III, to form superoxide [39]. In this study, we found that PMA induced aberrant angiogenesis, as shown by increased endothelial cell migration and tubule-like structure formation. The inflammatory angiogenic response triggered by PMA occurred through mechanisms that determine an increase in mtROS production. Indeed, PMA-induced endothelial migration and capillary morphogenesis were suppressed by preincubation with rotenone and antimycin A, inhibitors of the electron transport chain, respectively, at the level of mitochondrial complexes I and III, major sources of mtROS. In our model system, mtROS were boosted already at 30 min after PMA stimulation and their levels remained high after 16 h, suggesting mtROS as an initial and late player in PMA-induced endothelial dysfunction. Our results are in accordance with previous findings by Joo et al. [40] who reported that Mito-TEMPO, a specific mitochondrial antioxidant, inhibited the PMA-stimulated expression of endothelial adhesion molecules, implying an important role of mtROS in endothelial inflammation. About the role of HT in PMA-induced superoxide, we found that HT pretreatment decreased, in a concentration-dependent manner, superoxide production as well as membrane lipid peroxidation. Overall, our findings point out the key role of mtROS overproduction in aberrant inflammatory angiogenesis and suggest that the HT antiangiogenic effect could be ascribed to its protective action on endothelial mitochondria. It should be remarked that endothelial superoxide can be detoxified through the action of the mitochondrial MnSOD, a matrix-abundant and highly efficient enzyme that can convert superoxide to hydrogen peroxide, which is the more stable and less reactive form than superoxide [41]. Here, we found, in agreement with the reduced superoxide levels, that HT increased MnSOD activity in PMA-triggered endothelial cells. This result is in accordance with previous data showing HT effects on the activation and expression of several cytoprotective enzymes [12–14].

The primary function of mitochondria is to generate ATP by the oxidative process. ATP synthesis is driven via the transfer of electrons through complexes I to IV, generating a concentration gradient of protons across the inner mitochondrial membrane thus maintaining membrane potential. During stress conditions, electron transport and ATP synthesis often fail leading to the accumulation of ROS and mitochondrial dysfunction, along with a significant reduction of oxidative phosphorylation efficiency, due to membrane potential breakdown, ATP depletion, and uncoupled oxidative phosphorylation [42]. We found that associated with an overproduction of mtROS, PMA significantly reduced MMP as well as mitochondrial ATP synthesis in endothelial cells. The reduced production of mitochondrial ATP by PMA would seem in contrast with its proangiogenic action, since angiogenesis is a highly energetic process. However, our findings are in agreement with reports showing that the inhibition of mitochondrial ATP synthesis does not impair endothelial vessel sprouting [43] but has a critical role as a stress sensor in the dysfunctional endothelium [38]. According to decreased overproduction of mtROS, HT pretreatment attenuated the PMA-induced mitochondrial membrane depolarization in endothelial cells, and it recovered the lowered ATP levels by inducing FoF1-ATP synthase activity and the expression of catalytic subunit, ATP5*β*. These results highlight that HT can protect the endothelium against inflammation-induced injury, improving mitochondrial function and preventing mtROS overproduction.

To deepen the mechanisms of action underlying the HT endothelial mitochondrial protection, we studied the effects of HT on mitochondrial biogenesis, which has recently emerged as a potential therapeutic target to improve endothelial function [38]. Mitochondrial biogenesis is a highly regulated process requiring replication of mtDNA and expression of nuclear and mitochondrial genes [44–46]. The primary role is performed by PGC-1*α*, which activates NRF-1 to coordinate expression of nuclear genes required for biogenesis [47, 48]. PGC-1*α* also activates TFAM that is responsible for the transcriptional control of mtDNA [49]. It has been established that the increased expression of these factors modulates mitochondrial biogenesis in endothelial cells and plays a pivotal role in optimizing cellular mitochondrial function [50]. Our findings reveal that PMA decreased mitochondrial biogenesis by reducing the expression of PGC-1*α*, NRF-1, and TFAM as well as by decreasing the mtDNA copy number. The reduction of PMA-induced mitochondrial biogenesis is in accordance with the decreased ATP5*β* expression and mitochondrial ATP production. HT pretreatment restored PMA-reduced mitochondrial biogenesis, enhancing mtDNA content and PGC-1*α*, NRF-1, and TFAM expression. In line with this, HT also increased ATP5*β* expression and mitochondrial ATP production, resulting in an improved mitochondrial performance in endothelial cells under inflammatory conditions. Our observations are in agreement with previous *in vitro* findings about the HT ability to activate PGC-1*α* and to induce mitochondrial biogenesis in 3T3-L1 murine adipocytes and in ARPE-19 human retinal pigment epithelial cells [14, 51, 52]. Multiple lines of evidence have shown a multifactorial protection of PGC-1*α* in vascular health [46]. Vascular risk factors including hyperglycaemia significantly decrease PGC-1*α* protein expression and reduce the mitochondrial number as shown in the retina of diabetic patients and in retinal endothelial cells treated with high glucose [53, 54]. PGC-1*α* overexpression decreased endothelial inflammatory response [55], whereas PGC-1*α*-deficient mice displayed increased inflammatory markers in atherosclerotic plaques [56]. Moreover, PGC-1*α* can protect the endothelium through the inhibition of the redox-sensitive transcription factor NF-*κ*B, a crucial regulator of inflammation and endothelial dysfunction [55]. The increased PGC-1*α* expression by HT, shown in this study, is in accordance with previous findings, which indicate that HT reduced inflammatory angiogenesis by suppressing the activation of NF-*κ*B [20, 21]. Moreover, our findings further support other studies regarding the role of natural bioactive compounds as molecules capable of modulating mitochondrial biogenesis, MMP, mitochondrial electron transport chain, and ATP synthesis as well as mitochondrial oxidative status in endothelial cells [9, 33, 50, 57–59]. Noteworthy, HT protective effects were observed to occur at micromolar concentrations, which are physiologically relevant and nutritionally achievable, as HT is well absorbed in the small intestine and metabolized in the body after intake of virgin olive oil and/or olives [60].

**Figure 7:**
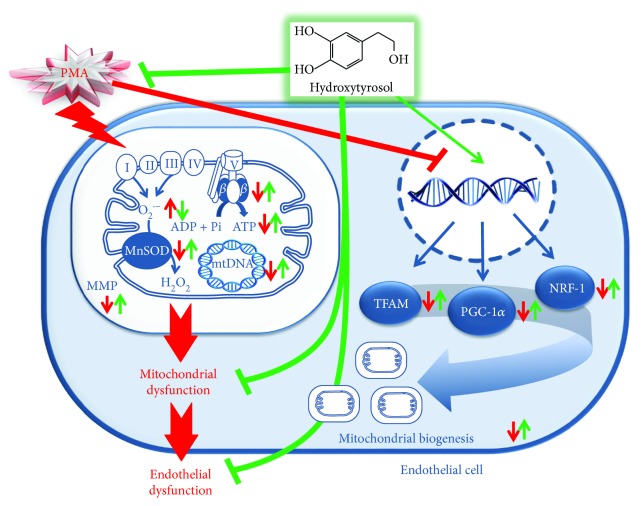
A schematic illustration of the molecular mechanism underlying HT protective effects on mitochondrial function in PMA-stimulated endothelial cells. Red line indicates PMA action, green line indicates HT effect, up arrow indicates upregulation, and down arrow indicates downregulation.

Taken together, these results indicate that HT could represent a mitochondria-targeting antioxidant nutrient in endothelial cells under inflammatory conditions, having a beneficial impact at a mitochondrial level by preventing the oxidative stress and improving the mitochondrial function and biogenesis (Figure 7).

## 5. Conclusion

This study demonstrates, for the first time, that HT can counteract inflammatory angiogenesis through the improvement of endothelial mitochondrial function and biogenesis. These properties point out HT as a mitochondrial nutrient targeting the inflamed endothelium and provide a new mechanism of action by which HT could prevent chronic degenerative pathologies, including cardiovascular diseases.

## Figures and Tables

**Table 1:** Primer sequence of real-time quantitative PCR.

Gene	Accession number	Primers (sequence 5′–3′)	Size (bp)
TNF-*α*	NM_000594.2	CCTGTGAGGAGGACGAACAT AGGCCCCAGTTTGAATTCTT	240
IL-1*β*	NM_000576.2	CTGTCCTGCGTGTTGAAAGA AGTTATATCCTGGCCGCCTT	228
VCAM-1	NM_001078.3	CATGGAATTCGAACCCAAAC CCTGGCTCAAGCATGTCATA	140
ICAM-1	NM_000201.2	AGACATAGCCCCACCATGAG CAAGGGTTGGGGTCAGTAGA	190
ATP5*β*	NM_001686	TGAGGGACTACCACCAATTC TTTCTGGCCTCTAACCAAGC	141
TFAM	NM_001270782.1	CCGAGGTGGTTTTCATCTGT ACGCTGGGCAATTCTTCTAA	147
NRF-1	NM_005011.3	CCGTTGCCCAAGTGAATTAT ACTGTAGCTCCCTGCTGCAT	181
PGC-1*α*	NM_013261.3	GCTGACAGATGGAGACGTGA TGCATGGTTCTGGGTACTGA	178
36B4	NM_001697.2	TCGACAATGGCAGCATCTAC ATCCGTCTCCACAGACAAGG	191
D-loop	AC_000022.2	GGTTCTTACTTCAGGGCCATC TGACCTTCATGCCTTGACGG	201
Gapdh	NG_007073.2	ATGCCTTCTTGCCTCTTGTC CATGGGTGGAATCATATTGG	245

## Supplementary Materials

Supplementary Materials Supplementary material includes the experimental procedure to detect nitric oxide levels, and the Supplementary Figure 1 shows the HT effects on PMA-induced nitric oxide production. Supplementary Figure 1: HT effects on PMA-induced nitric oxide production. HUVEC were pretreated with HT (1–30 *μ*mol/L) or vehicle (control, CTR) for 1 h and stimulated by PMA (10 nmol/L) for 16 h; then, nitric oxide was quantified as its stable oxidation product, nitrite, in the supernatant, by using the nitrite assay kit. Data represent three independent experiments, and they are expressed as means ± SD.
