# Supplementary material for: Hydroxytyrosol Ameliorates Endothelial Function under Inflammatory Conditions by Preventing Mitochondrial Dysfunction
Source: Oxid Med Cell Longev. 2018 Apr 18;2018:9086947. doi: 10.1155/2018/9086947 (PMC5932486; doi:10.1155/2018/9086947)
Supplement: Supplementary Materials — Supplementary material includes the experimental procedure to detect nitric oxide levels, and the Supplementary Figure 1 shows the HT effects on PMA-induced nitric oxide production. Supplementary Figure 1: HT effects on PMA-induced nitric oxide production. HUVEC were pretreated with HT (1–30 μmol/L) or vehicle (control, CTR) for 1 h and stimulated by PMA (10 nmol/L) for 16 h; then, nitric oxide was quantified as its stable oxidation product, nitrite, in the supernatant, by using the nitrite assay kit. Data represent three independent experiments, and they are expressed as means ± SD. [file 9086947.f1.pdf]

Hydroxytyrosol ameliorates endothelial function under inflammatory conditions by preventing mitochondrial dysfunction

Nadia Calabriso[a], Antonio Gnoni[b], Eleonora Stanca[c], Alessandro Cavallo[c], Fabrizio Damiano[c], Luisa Siculella [c*], Maria Annunziata Carluccio[a**]

[a]National Research Council - Institute of Clinical Physiology, Lecce, Italy
[b]Department of Basic Medical Sciences, Neurosciences and Sense Organs, University of Bari "Aldo Moro", Bari, Italy
[c]Laboratory of Biochemistry and Molecular Biology, Department of Biological and Environmental Sciences and Technologies, University of Salento, Lecce, Italy

[*]Correspondence to: Luisa Siculella
Department of Biological and Environmental Sciences and Technologies, University of Salento, 73100 Lecce, Italy
E-mail address: luisa.siculella@unisalento.it

[**]Correspondence to: Maria Annunziata Carluccio
National Research Council - Institute of Clinical Physiology, 73100 Lecce, Italy
E-mail address: maria.carluccio@ifc.cnr.it

Supplementary Materials

Supplementary Methods

Determination of endothelial nitric oxide production

Production of nitric oxide (NO) by HUVEC was measured as its stable oxidation product, nitrite, determined in the supernatant, using colorimetric assay kit (Cayman), based on Griess reagents (sulfanilamide and naphthalene-ethylenediamine dihydrochloride), according to manufacturer's instructions.

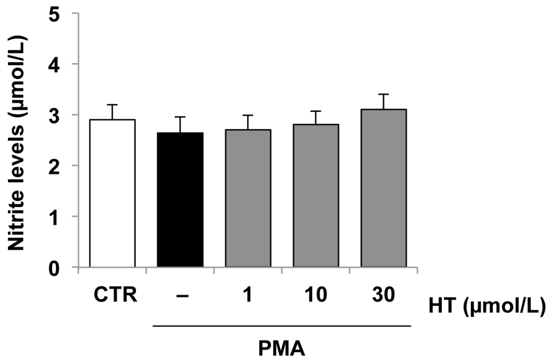

Supplementary Figure 1: HT effects on PMA-induced nitric oxide production

HUVEC were pre-treated with HT (1-30 µmol/L) or vehicle (control, CTR) for 1 h and stimulated by PMA (10 nmol/L) for 16 h, then nitric oxide was quantified as its stable oxidation product, nitrite, in the supernatant, by using nitrite assay kit. Data represent three independent experiments and, they are expressed as means ± SD.
